# Molecular Insights into How the Dimetal Center in Dihydropyrimidinase Can Bind the Thymine Antagonist 5-Aminouracil: A Different Binding Mode from the Anticancer Drug 5-Fluorouracil

**DOI:** 10.1155/2022/1817745

**Published:** 2022-02-14

**Authors:** En-Shyh Lin, Ren-Hong Luo, Ya-Ching Yang, Cheng-Yang Huang

**Affiliations:** ^1^Department of Beauty Science, National Taichung University of Science and Technology, Taichung City, Taiwan; ^2^School of Biomedical Sciences, Chung Shan Medical University, Taichung City, Taiwan; ^3^Department of Medical Research, Chung Shan Medical University Hospital, Taichung City, Taiwan

## Abstract

Dihydropyrimidinase (DHPase) is a key enzyme for pyrimidine degradation. DHPase contains a binuclear metal center in which two Zn ions are bridged by a posttranslationally carbamylated lysine. DHPase catalyzes the hydrolysis of dihydrouracil to *N*-carbamoyl-*β*-alanine. Whether 5-aminouracil (5-AU), a thymine antagonist and an anticancer drug that can block DNA synthesis and induce replication stress, can interact with DHPase remains to be investigated. In this study, we determined the crystal structure of *Pseudomonas aeruginosa* DHPase (PaDHPase) complexed with 5-AU at 2.1 Å resolution (PDB entry 7E3U). This complexed structure revealed that 5-AU interacts with Zn*α* (3.2 Å), Zn*β* (3.0 Å), the main chains of residues Ser289 (2.8 Å) and Asn337 (3.3 Å), and the side chain of residue Tyr155 (2.8 Å). These residues are also known as the substrate-binding sites of DHPase. Dynamic loop I (amino acid residues Pro65-Val70) in PaDHPase is not involved in the binding of 5-AU. The fluorescence quenching analysis and site-directed mutagenesis were used to confirm the binding mode revealed by the complexed crystal structure. The 5-AU binding mode of PaDHPase is, however, different from that of 5-fluorouracil, the best-known fluoropyrimidine used for anticancer therapy. These results provide molecular insights that may facilitate the development of new inhibitors targeting DHPase and constitute the 5-AU interactome.

## 1. Introduction

Pyrimidine bases are essential for the replication of genetic information, cellular metabolism, and cell growth in all biological systems [1]. Uracil derivatives, especially 5-substituted uracils, play a significant role in pharmacological activities, such as antiviral [2], anticancer drugs [3], antibacterial [4], anti-inflammatory [5, 6], and antitumor activities [7–9]. For example, 5-fluorouracil (5-FU) is an FDA-approved drug with a remarkable therapeutic effect for the systemic treatment of cancers of the gastrointestinal tract, breast, head, and neck in the clinic [8]. As a thymine antagonist possessing anticancer activities, 5-aminouracil (5-AU) can block DNA synthesis and induce replication stress [10, 11]. In addition, recent findings indicate that microbiota can modulate the host response to these chemotherapeutic drugs [12]. Thus, the whole interactome of these five-substituted uracil drugs should be determined for detailed clinical pharmacokinetic and toxicity analyses.

**Figure 1:**
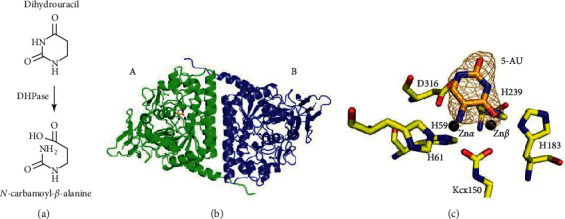
The structure of PaDHPase complexed with 5-AU. (a) The physiological reaction of DHPase. (b) The complexed structure of PaDHPase with 5-AU. The monomers are colored differently. 5-AU was found only in monomer A and not in monomer B. (c) The active site of PaDHPase. The binuclear metal center assembled by residues His59, His61, carbamylated Lys150 (Kcx150), His183, His239, and Asp316 in PaDHPase is essential for catalytic activity. The light orange mesh is the 2Fo-Fc electron density map from the 5-AU contoured at 1*σ* with a 1.6 Å carve radius. Both two zinc ions were involved in binding 5-AU.

Dihydropyrimidinase (DHPase) [13] is a key enzyme for pyrimidine degradation (Figure 1(a)). DHPase contains a binuclear metal center in which two Zn ions are bridged by a posttranslationally carbamylated lysine (Kcx) [14–18]. DHPase catalyzes the hydrolysis of dihydrouracil (DHU) to *N*-carbamoyl-*β*-alanine. Including a Kcx, the dimetal center (Zn*α*/Zn*β*) of DHPase consists of four His and one Asp. The conserved substrate binding sites of DHPase are Ser, Asn, and Tyr [19]. Of these residues, Ser and Asn interact with the substrate DHU through the backbone of DHPase. Despite the interactions via the main chain, mutations of these residues still lead to severely impaired enzymes [16]. Loss of the Zn*β* ion causes Kcx to no longer be carbamylated in mono-Zn DHPase [14]. On the basis of an analysis of the amino acid sequences, DHPase was suggested to be a member of the cyclic amidohydrolase family [20, 21]. The cyclic amidohydrolase family also includes dihydroorotase [22–28], allantoinase [28–30], hydantoinase [31, 32], and imidase [33–35]. These metal-dependent enzymes catalyze the hydrolysis of the cyclic amide bond of each substrate in the metabolism of purines and pyrimidines. Some of these amidohydrolases are known to be anticancer [24, 36–38], antimicrobial [16, 27, 39], and antimalarial targets [40, 41] because of their involvement in the key reactions of nucleotide synthesis. Therefore, new inhibitors should be found, and their binding modes should be investigated for chemotherapeutic drug development.

5-FU-associated toxicity was reported [42]. After treatment of 5-FU for anticancer therapy, the asymptomatic patients with DHPase deficiency suffered from severe toxicity, including death [43]. A recent report revealed that DHPase is a novel 5-FU-binding protein [44]. Whether and how other five-substituted uracil drugs such as 5-AU can bind to DHPase remains to be elucidated. In the present study, we found that 5-AU can bind to *Pseudomonas aeruginosa* DHPase (PaDHPase). We determined the crystal structure of the PaDHPase complex with 5-AU at a 2.1 Å resolution (PDB entry 7E3U). Given the structural resemblance between 5-FU and 5-AU, one might conclude that the mode of 5-FU bound by PaDHP must be similar to that of 5-AU. However, we found that their binding modes are different. The fluorescence quenching method and site-directed mutagenesis were also used to confirm the binding mode of 5-AU to PaDHPase revealed by the complex structure. We think that the determination of this complex structure can extend the knowledge of the 5-AU interactome and benefit anticancer development.

## 2. Materials and Methods

### 2.1. Expression and Purification of PaDHPase

The expression vector pET21b-PaDHPase [16] was transformed into *Escherichia coli* BL21 (DE3) cells and grown in LB medium at 37°C. The overexpression was induced by incubating with 1 mM isopropyl thiogalactopyranoside for 9 h. Recombinant PaDHPase [36] was purified from the soluble supernatant by using Ni^2+^-affinity chromatography (HiTrap HP; GE Healthcare Bio-Sciences). The recombinant protein was eluted with a linear imidazole gradient and dialyzed against a dialysis buffer (20 mM Tris-HCl and 0.1 M NaCl, pH 7.9; Buffer A).

### 2.2. Preparation of Mono-Zn PaDHPase (Zn*α*-PaDHPase)

The mono-Zn PaDHPase was prepared using the protocol described previously [45]. Purified PaDHPase was dialyzed against a chelating buffer (50 mM MES, 50 mM EDTA, and 15 mM 8-HQSA, pH 6.5; Buffer B) at room temperature for 3 days. The resultant enzyme solution was then dialyzed against Buffer A.

### 2.3. Site-Directed Mutagenesis

The PaDHPase mutants were generated according to the QuikChange Site-Directed Mutagenesis kit protocol (Stratagene; La Jolla, CA, USA). The wild-type plasmid pET21b-PaDHPase was used as a template [16]. The presence of the mutation was verified by DNA sequencing in each construct. The recombinant mutant proteins were purified using the protocol for the wild-type PaDHPase by Ni^2+^-affinity chromatography (Table 1).

### 2.4. Crystallography

The optimal protein concentration for crystallization of the PaDHPase complex, determined by the Pre-Crystallization Test (Hampton), was 10 mg/mL. Crystals were grown at room temperature by hanging drop vapor diffusion in 16% PEG 8000, 100 mM HEPES, 200 mM calcium acetate, and 200 *μ*M 5-AU, at pH 7.5. Data were collected using an ADSC Quantum-315r CCD area detector at SPXF beamline BL13C1 at the National Synchrotron Radiation Research Center (NSRRC; Hsinchu, Taiwan). The structure of the 5-AU-PaDHPase complex was solved to 2.1 Å resolution with the molecular replacement software Phaser-MR [46] using PaDHPase (PDB entry 5E5C) [15] as a model (Table 2). The data were indexed and scaled using HKL-2000 [47]. Models were built and refined with PHENIX [48] and Coot [49]. Coordinate and structure factor files have been deposited in the Protein Data Bank (PDB entry 7E3U).

### 2.5. Determination of the Dissociation Constant (K_d_)

Through the fluorescence quenching analysis, the *K*_*d*_ value of the purified PaDHPase was determined [44]. An aliquot of 5-AU was added into the solution containing PaDHPase (0.8 *μ*M) and 50 mM HEPES at pH 7.0. The decrease in the intrinsic fluorescence of PaDHPase was measured at 336 nm upon excitation at 279 nm and 25°C with a spectrofluorimeter (Hitachi F-2700; Hitachi High-Technologies, Japan). The *K*_d_ was obtained using the equation: Δ*F* = Δ*F*_max_ − *K*_*d*_(Δ*F*/[5-AU]).

## 3. Results and Discussion

### 3.1. Crystallization of the PaDHPase-5-AU Complex

PaDHPase crystals can be obtained by hanging drop vapor diffusion in 28% PEG 6000, 100 mM HEPES, and 200 mM lithium acetate at pH 7.5 [15]. Soaking and cocrystallization of PaDHPase with 5-AU under these conditions were attempted but were unsuccessful. After crystallization screening, the crystals of the PaDHPase-5-AU complex appeared at room temperature in 16% PEG 8000, 100 mM HEPES, 200 mM calcium acetate, and 200 *μ*M 5-AU, at pH 7.5. These crystals were used for determining the complex structure of PaDHPase.

### 3.2. Structure of PaDHPase in Complex with 5-AU

The crystals of the 5-AU-PaDHPase complex belong to space group P3_1_2_1_ with cell dimensions of *a* = 112.67, *b* = 112.67, and *c* = 161.43 Å. The complexed crystal structure of PaDHPase with 5-AU was determined at a 2.1 Å resolution (Table 2). Two monomers of PaDHPase were found in the asymmetric unit (Figure 1(b)). Consistently, PaDHPase functions as a dimer [15]. The electron density of 5-AU was well defined and indicated the presence of 5-AU in the active site of PaDHPase (Figure 1(c)). The orientation of 5-AU could be easily distinguished on the basis of the location of the substituent. However, only one 5-AU molecule was found in the active site of one of the PaDHPase dimers. This is also the case for 5-FU bound to PaDHPase [44]. The binding of 5-AU does not influence the overall structure of PaDHPase. Similar to the apo form, the global architecture of the 5-AU-complexed PaDHPase monomer (subunit A) revealed a TIM-barrel structure embedding the catalytic dimetal center (Zn*α* and Zn*β*) and a *β*-sandwich domain, consisting of 17 *α*-helices, 19 *β*-sheets, 2 Zn ions, and 1 5-AU molecule. Both of the two zinc ions are involved in binding 5-AU. The dimetal center in the PaDHPase-5-AU complex consisted of His59, His61, Kcx150, His183, His239, and Asp316 still self-assembles (Figure 1(c)). Lys150 remains carbamylated (Kcx150) regardless of 5-AU binding.

### 3.3. 5-AU Binding Mode

**Figure 2:**
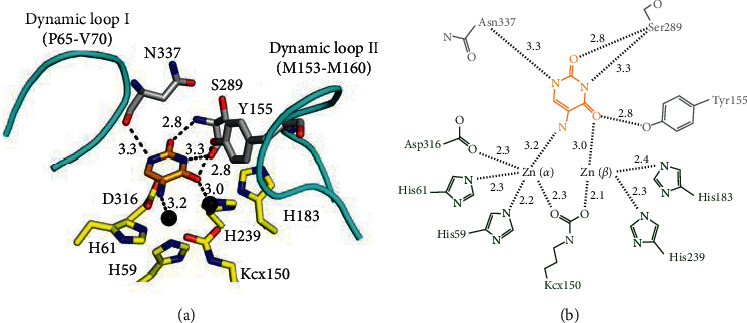
5-AU binding mode. (a) The interactions of PaDHPase with 5-AU. Two metal ions (black spheres) and the substrate binding sites (gray) in PaDHPase were involved in 5-AU (light orange) binding. Dynamic loops are colored in cyan. 5-AU interacted with the main chains of residues Ser289 and Asn337 and the side chain of residue Tyr155. The distances are shown on dotted lines. (b) 5-AU interacted with Zn*α*, Zn*β*, Ser289, Asn337, and Tyr155. Residues (His59, His61, Kcx150, His183, His239, and Asp316) required for metal binding are colored in green.

As a uracil derivative possessing many chemotherapeutic and pharmacological activities [11], 5-AU was identified as a ligand bound by PaDHPase in this study. The 5-AU binding mode of PaDHPase was demonstrated by the complexed crystal structure. On the basis of our crystallographic analysis, various interactions between 5-AU and PaDHPase were examined (Figure 2(a)). Residues Tyr155, Ser289, and Asn337 of PaDHPase, crucial for substrate binding [17, 19], are also involved in 5-AU binding (Figure 2(b)). 5-AU interacts with Zn*α* (3.2 Å), Zn*β* (3.0 Å), the main chains of residues Ser289 (2.8 Å) and Asn337 (3.3 Å), and the side chain of residue Tyr155 (2.8 Å). Dynamic loop I (amino acid residues Pro65-Val70) in PaDHPase is not involved in the binding of 5-AU.

### 3.4. Structural Comparison of the Active Sites between the 5-AU Bound State and the 5-FU Bound State of PaDHPase

**Figure 3:**
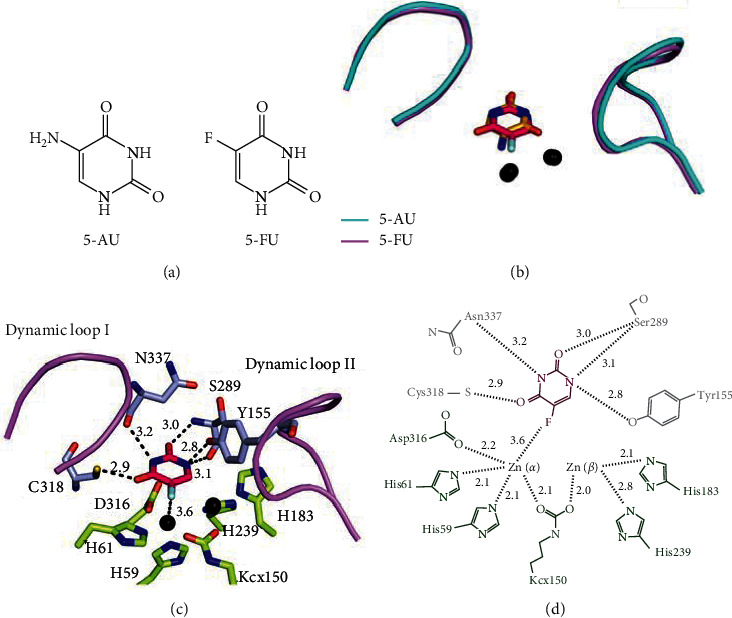
Comparison between the 5-AU and 5-FU binding modes. (a) The structure of 5-AU and 5-FU. (b) The superimposed structures of the 5-AU and 5-FU bound states. The conformation of the dynamic loops in both binding states is similar, but the orientations between 5-AU and 5-FU (hot pink) are different. (c) The interactions of PaDHPase with 5-FU. Zn*α* and the substrate binding sites (gray) in PaDHPase were involved in 5-FU binding. Dynamic loops are colored in light pink. 5-FU interacted with the main chains of residues Ser289 and Asn337 and the side chains of residues Tyr155 and Cys318. The distances are shown on dotted lines. (d) 5-FU interacted with Zn*α*, Ser289, Cys318, Asn337, and Tyr155. Residues (His59, His61, Kcx150, His183, His239, and Asp316) required for metal binding are colored in green.

Recently, the crystal structure of PaDHPase in complex with the anticancer drug 5-FU was reported [44]. 5-FU is the best-known fluoropyrimidine used to target the enzyme thymidylate synthase in anticancer therapy [3, 8]. Given the structural similarity between 5-AU and 5-FU (Figure 3(a)), one might conclude that their binding mode by PaDHPase must be similar. The dynamic loops of PaDHPase extend toward the active site when either 5-FU or 5-AU is bound (Figure 3(b)). However, we found that 5-AU and 5-FU binding poses to PaDHPase are different in terms of orientation (Figure 3(b)) and binding residues (Figure 3(c)). The side chain of residue Cys318 in PaDHPase (Figure 3(d)) is involved in binding 5-FU (2.9 Å) but not 5-AU (Figure 2). In addition, the binding contributions from the metal ions in PaDHPase are different. Zn*β* is not involved in binding 5-FU [44], whereas both Zn ions interact with 5-AU (Figure 2). Thus, we concluded that the binding mechanisms of the anticancer drugs 5-FU and 5-AU to PaDHPase are different.

### 3.5. 5-AU Binding Analysis

**Figure 4:**
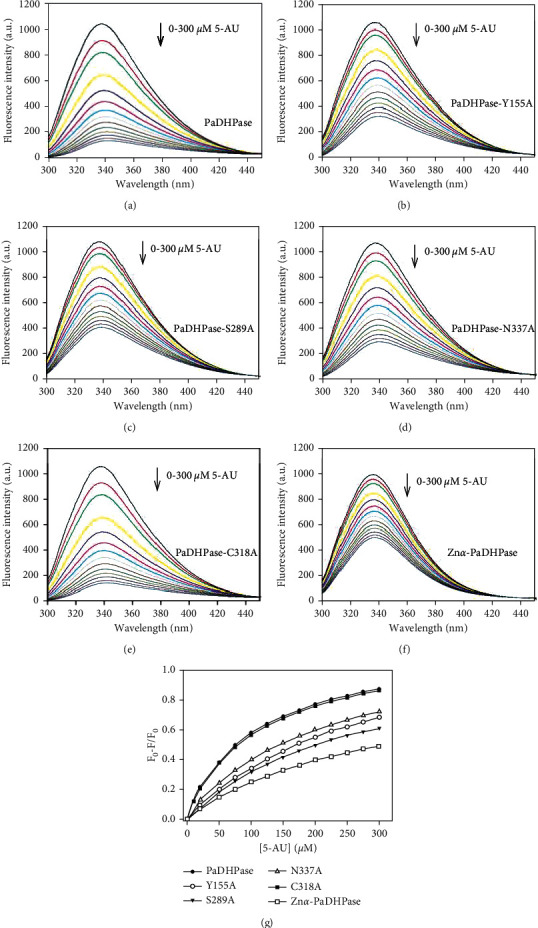
Fluorescence titration of PaDHPase with 5-AU. (a) The fluorescence emission spectra of PaDHPase with 5-AU of different concentrations (0–300 *μ*M). The decrease in intrinsic fluorescence of protein was measured at 336 nm upon excitation at 279 nm with a spectrofluorimeter. The fluorescence intensity emission spectra of PaDHPase significantly quenched with 5-AU. (b) The fluorescence emission spectra of PaDHPase-Y155A with 5-AU of different concentrations (0–300 *μ*M). (c) The fluorescence emission spectra of PaDHPase-S289A with 5-AU. (d) The fluorescence emission spectra of PaDHPase-N337A with 5-AU. (e) The fluorescence emission spectra of PaDHPase-C318A with 5-AU. (f) The fluorescence emission spectra of Zn*α*-PaDHPase with 5-AU. (g) An aliquot amount of 5-AU was added to the enzyme solution for determining the *K*_d_. The *K*_d_ was obtained by the equation: Δ*F* = Δ*F*_max_ − *K*_d_(Δ*F*/[5-AU]). Data points are an average of 2–3 determinations within 10% error.

Prior to this study, whether 5-AU could bind to DHPase remained unknown. To confirm if PaDHPase is capable of binding 5-AU, we used the fluorescence quenching method to determine the binding ability of PaDHPase (Figure 4). Quenching is a complex formation process that decreases the fluorescence intensity of protein. The fluorescence emission spectra of the wild-type PaDHPase was significantly quenched with 5-AU (Figure 4(a)). PaDHPase displayed strong intrinsic fluorescence with a peak wavelength of 336 nm when excited at 279 nm. When 5-AU was titrated into the PaDHPase solution, the intrinsic fluorescence of the protein was progressively quenched. Upon the addition of 300 *μ*M 5-AU, the intrinsic fluorescence of PaDHPase was quenched by 87.3%. Adding 5-AU resulted in a red shift in the PaDHPase emission wavelength (∼6.5 nm; *λ*_max_ from 336.5 to 343 nm). These observations indicated that PaDHPase can form a stable complex with 5-AU. As determined through the titration curve, the dissociation constant (*K*_d_) of PaDHPase bound to 5-AU is 97.7 ± 2.0 *μ*M. Based on the *K*_d_ value of PaDHPase bound to 5-FU (133.2 ± 8.5 *μ*M) [44], PaDHPase may prefer binding to 5-AU over 5-FU.

### 3.6. Structure-Based Mutational Analysis

The complexed structure revealed Tyr155, Ser289, and Asn337 of PaDHPase as the 5-AU binding sites (Figure 2). 5-AU interacts with the main chains of residues Ser289 and Asn337 and the side chain of residue Tyr155. To investigate the contribution of individual amino acid residues to 5-AU binding, alanine substitution mutants (Table 2) were constructed and analyzed by fluorescence quenching (Figures 4(b)–4(d)). We found that 300 *μ*M 5-AU quenched the intrinsic fluorescence of mutants Y155A, S289A, and N337A by 68.6%, 61.6%, and 72.4%, respectively. The *K*_d_ values of Y155A, S289A, and N337A bound to 5-AU were reduced to 247.1 ± 4.0, 256.0 ± 3.2, and 192.1 ± 8.0 *μ*M, respectively. Accordingly, Ser289 is the most effective one of these residues for 5-AU binding.

Structurally, Cys318 is involved in 5-FU [44] but not 5-AU. For comparison, the C318A mutant was also analyzed by fluorescence quenching (Figure 4(e)). We found that 300 *μ*M 5-AU quenched the intrinsic fluorescence of C318A by 86.8%. The *K*_d_ value of C318A was determined as 103.3 ± 3.1 *μ*M. The abilities of the wild-type PaDHPase and the C318A mutant to bind 5-AU are approximately equal. Thus, Cys318 is not essential for 5-AU binding.

### 3.7. Role of Zn*β* Ion in 5-AU Binding

On the basis of the complex structure of 5-AU-PaDHPase, the Zn*β* ion is suggested to be critical for 5-AU binding (Figure 2). To confirm the role of Zn*β* in 5-AU binding and to compare the binding contribution with other mutant proteins (Table 3), we produced mono-Zn PaDHPase (Zn*α*-PaDHPase) for binding analysis. Our crystal structure previously revealed that this mono-Zn enzyme [45] only contains a Zn*α* ion in the active site of PaDHPase. The binding of 5-AU to Zn*α*-PaDHPase was also analyzed by the fluorescence quenching method (Figure 4(f)). Upon the addition of 300 *μ*M 5-AU, the intrinsic fluorescence of Zn*α*-PaDHPase was quenched by 49.3%. The *K*_d_ value of Zn*α*-PaDHPase for 5-AU binding was calculated to be 281.5 ± 9.0 *μ*M from the titration curve (Figure 4(g)). Based on the *K*_d_ values, the strength of complex formation with 5-AU followed the following order: the wild-type enzyme ≈ C318A > N337A > Y155A ≈ S289A > Zn*α*-PaDHPase (Table 3).

### 3.8. The 5-AU Structural Interactome

**Figure 5:**
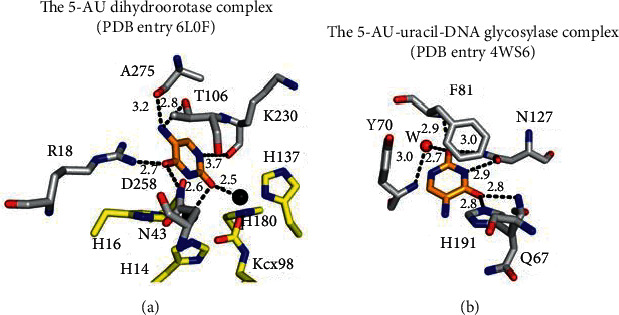
5-AU binding modes. (a) The interactions of dihydroorotase with 5-AU. Two metal ions (black spheres) and the binding sites (gray) in dihydroorotase are involved in 5-AU (light orange) binding. (b) The interactions of uracil-DNA glycosylase with 5-AU.

Metabolic reprogramming allows the cancer cells to rapidly proliferate, resist chemotherapies, invade, metastasize, and survive in a nutrient-deprived microenvironment [1]. Many uracil derivatives have long been used for anticancer treatment, such as 5-FU, the most commonly used pyrimidine-based antimetabolite targeting thymidylate synthase [3, 8]. 5-AU also possesses potent anticancer activities that can block DNA synthesis and induce replication stress [11]. 5-AU can significantly induce biphasic interphase-mitotic (IM) cells [10]. Although 5-AU and 5-FU are similar uracil derivatives, no induction of IM cells was detected even using excess 5-FU [10]. Therefore, 5-AU and 5-FU might somehow induce different cellular effects. In this study, we found different binding modes of PaDHPase between 5-AU and 5-FU, and whether the different binding modes can produce distinct signaling pathways has not yet been elucidated. We noticed that other enzymes also respond to 5-AU by binding and/or inhibition. Three 5-AU-complexed protein structures are available in the PDB for comparison: DHPase (this study), uracil-DNA glycosylase (PDB entry 4WS6), and dihydroorotase (PDB entry 6L0F). These three enzymes bind 5-AU via different binding environments (Figure 5). For example, uracil-DNA glycosylase binds 5-AU via Gln67, Tyr70, Phe81, Asn127, and His191 [50]. Dihydroorotase binds 5-AU via Arg18, Asn43, Thr106, Lys230, and Ala275 [25]. These interactions involving 5-AU binding, including that of PaDHPase-5-AU, are different. Further structural studies are needed to understand 5-AU's binding mechanisms for building the structural interactome for detailed clinical pharmacokinetics and toxicity analyses.

## 4. Conclusion

We identified that PaDHPase can bind 5-AU with a *K*_d_ value of 97.7 *μ*M (Table 3 and Figure 4). The 5-AU binding mode of PaDHPase, different from that of 5-FU, was determined through structural evidence (Figure 1) and mutational analysis (Table 3). This structure provides molecular insights into how the dimetal center in PaDHPase can bind 5-AU (Figure 2). Further research can directly focus on revisiting the role of DHPase in anticancer therapy [42, 43, 51].

## Figures and Tables

**Table 1:** Primers used for construction of plasmids.

Oligonucleotide	Primer
Y155A-N	CACTTCATGGCCGCCAAGAACGCCATCATGGCC
Y155A-C	CATGATGGCGTTCTTGGCGGCCATGAAGTGCTT
S289A-N	GGCTACGTGATGGCCCCGCCGTTCCGTCCCGTC
S289A-C	GGGACGGAACGGCGGGGCCATCACGTAGCCGGC
C318A-N	CCGCCACCGACCACGCCTGCTTCTGCGCCGAGC
C318A-C	CGCAGAAGCAGGCGTGGTCGGTGGCGGTGGTAT
N337A-N	TTCAGCAAGATTCCCGCTGGCACGGCCGGCATC
N337A-C	GCCGGCCGTGCCAGCGGGAATCTTGCTGAAGTC

These plasmids were verified by DNA sequencing. Underlined nucleotides indicate the designated site for mutation or the restriction site.

**Table 2:** Data collection and refinement statistics.

Data collection	
Crystal	PaDHP-5AU
Wavelength (Å)	1.0
Resolution (Å)	30–2.16
Space group	P3_1_2_1_
Cell dimension *a*, *b*, *c* (Å)/*β* (°)	112.67, 112.67, 161.43/90
Redundancy	4.9 (4.9)
Completeness (%)	99.9 (99.9)
<*I*/*σI*>	23.7 (3.1)
CC_1/2_	0.965(0.866)

*Refinement*	
Resolution (Å)	29.01–2.16
No. of reflections	64183
*R*_work_/*R*_free_	0.185/0.229

*No. of atoms*	
Ligands	1
Protein	954
Zinc	4
Water	459

*r.m.s deviations*	
Bond lengths (Å)	0.008
Bond angles (°)	0.893

*Ramachandran plot*	
Favored (%)	95.45
Allowed (%)	3.7
Outliers (%)	0.85
PDB entry	7E3U

Values in parentheses are for the highest resolution shell. CC_1/2_ is the percentage of correlation between the intensities of random half-data sets.

**Table 3:** Binding parameters of PaDHPase to 5-AU.

PaDHPase	*λ*_max_ (nm)	*λ*_em_ shift (nm)	Quenching (%)	*K*_d_ value (*μ*M)
PaDHPase	From 336.5 to 343	6.5	87.3	97.7 ± 2.0
PaDHPase-Y155A	From 336.5 to 342	6.0	68.6	247.1 ± 4.0
PaDHPase-S289A	From 337 to 339	2.0	61.6	256.0 ± 3.2
PaDHPase-N337A	From 336 to 339.5	3.5	72.4	192.1 ± 8.0
PaDHPase-C318A	From 336 to 342	6.0	86.8	103.3 ± 3.1
Zn*α*-PaDHPase	From 335 to 336.5	1.5	49.3	281.5 ± 9.0

The decrease in the intrinsic fluorescence of ScDHOase was measured with a spectrofluorimeter (Hitachi F-2700; Hitachi High-Technologies, Japan). The *K*_d_ was obtained using the equation: Δ*F* = Δ*F*_max_ − *K*_d_(Δ*F*/[5-AU]).

## Data Availability

Atomic coordinates and related structure factors were deposited in the PDB with accession code 7E3U. All the data used to support the findings of this study are available from the corresponding author upon request.
